# Strawberry Accessions with Reduced *Drosophila suzukii* Emergence From Fruits

**DOI:** 10.3389/fpls.2016.01880

**Published:** 2016-12-21

**Authors:** Xiaoyun Gong, Lasse Bräcker, Nadine Bölke, Camila Plata, Sarah Zeitlmayr, Dirk Metzler, Klaus Olbricht, Nicolas Gompel, Martin Parniske

**Affiliations:** ^1^Department of Genetics, Faculty of Biology, Ludwig-Maximilians-Universität München (LMU Munich)Planegg-Martinsried, Germany; ^2^Department of Evolutionary Ecology, Faculty of Biology, Ludwig-Maximilians-Universität München (LMU Munich)Planegg-Martinsried, Germany; ^3^Institute of Botany, Department of Biology, Faculty of Science, Technische Universität DresdenGermany; ^4^Department of Quantitative Genetics, Faculty of Biology, Ludwig-Maximilians-Universität München (LMU Munich)Planegg-Martinsried, Germany; ^5^Hansabred GmbH & Co. KGDresden, Germany; ^6^Albrecht Daniel Thaer-Institute, Humboldt-Universität zu BerlinBerlin, Germany

**Keywords:** *Drosophila suzukii*, *Fragaria*, plant–insect interactions, plant disease resistance, soft fruits, horticulture

## Abstract

*Drosophila suzukii* is threatening soft fruit production worldwide due to the females’ ability to pierce through the intact skin of ripe fruits and lay eggs inside. Larval consumption and the associated microbial infection cause rapid fruit degradation, thus drastic yield and economic loss. Cultivars that limit the proliferation of flies may be ideal to counter this pest; however, they have not yet been developed or identified. To search for potential breeding material, we investigated the rate of adult *D. suzukii* emergence from individual fruits (fly emergence) of 107 accessions of *Fragaria* species that had been exposed to egg-laying *D. suzukii* females. We found significant variation in fly emergence across strawberries, which correlated with accession and fruit diameter, and to a lesser extent with the strawberry species background. We identified accessions with significantly reduced fly emergence, not explained by their fruit diameter. These accessions constitute valuable breeding material for strawberry cultivars that limit *D. suzukii* spread.

## Introduction

The spotted wing fly, *Drosophila suzukii*, is one of the most serious pests in soft fruit production, attacking several fruits of agricultural importance such as strawberries, raspberries, blueberries, grapes, blackberries and cherries. A key feature of this species is the serrated ovipositor of *D. suzukii* females, which enables them to pierce ripening fruits and lay eggs inside the flesh (Atallah et al., 2014). In contrast, most closely related species deposit their eggs in decaying fruits. The infestation by *D. suzukii* typically leads to complete loss of the fruit. In addition to the larvae consuming the fruit flesh, the wound created by the fly’s ovipositor constitutes an entry point for bacteria and fungi that lead to fruit rotting and decay (Cini et al., 2012). With an average generation time of ca. 4 weeks under favorable conditions, this pest can produce 7–15 generations per cropping season if uncontrolled, resulting in an explosive population growth (Cini et al., 2012; Lin et al., 2014). Yield losses caused by *D. suzukii* attacks can vary from negligible to over 90% (Bolda et al., 2010; Berry, 2012). The resulting economic loss, due to yield loss and pest management expenses was estimated to be over 500 million dollars yearly for the USA alone (Bolda et al., 2010; Goodhue et al., 2011).

The proposed origin of *D. suzukii* is East Asia (Adrion et al., 2014) and *D. suzukii* has been observed in China, Japan, Myanmar, and Thailand (Kanzawa, 1936; Cini et al., 2014). Since the first reported outbreaks outside of East Asia in 2008, it has, however, considerably spread across the world. The outbreak of *D. suzukii* has first been recorded from Hawaii (USA; Kaneshiro, 1983), California (USA), Spain, and Italy, then quickly spread across North America (Walsh et al., 2011), and arrived in Mexico (Hauser, 2011) and Brazil (Deprá et al., 2014); and it has simultaneously invaded most European countries: north, up to the UK and east, to Hungary (Calabria et al., 2012; Cini et al., 2014). This rapid geographical expansion of *D. suzukii* turned this pest into a novel global threat to soft fruit production.

Current control methods, already implemented or under development include the use of fly traps, field sanitation, chemical sprays (Walsh et al., 2011) and biocontrol with parasitoid or predators, or both (Chabert et al., 2012; Cuthbertson et al., 2014; Haye et al., 2016).

The development and deployment of cultivars that do not support the propagation of *D. suzukii* may be an effective approach to reduce the agricultural damage caused by *D. suzukii*, as an alternative to current chemical control and laborious field management. Previous studies have investigated traits that contribute to the susceptibility of fruits to *D. suzukii* infestation, including fruit firmness or penetration force (Burrack et al., 2013; Kinjo et al., 2013; Ioriatti et al., 2015; Lee et al., 2015), pH of the fruits (Lee et al., 2015), and brix level of the fruits (Lee et al., 2011, 2015). However, little has been done to search for natural variation in susceptibility/resistance within genotypes of one fruit crop, which would allow identification of resistant genotypes and genetic determinants of fruit traits that can later be exploited for cultivar development.

Compared to other soft fruits of agricultural and economical importance under attack, many of which are tree fruits, strawberry (genus *Fragaria*) is genetically amenable because of its relatively short generation time and small genome size (Hummer et al., 2011). Genome sequences of diploid *Fragaria* and octoploid *Fragaria* × *ananassa* species are already published (Shulaev et al., 2011; Hirakawa et al., 2013). Moreover, strawberries offer extensive phenotypic diversity sampled in breeders’ collections. The germplasm collection established by Professor Günter Staudt (hereafter referred to as the “Professor Staudt Collection”), for instance, is maintained at the strawberry breeding company Hansabred in Dresden, Germany and consists of 520 accessions of 25 known *Fragaria* species and natural hybrids (Olbricht et al., 2014).

Here we have screened the fruits of 107 accessions of the “Professor Staudt Collection” for their ability to support *D. suzukii* development. Our goal was to identify potential germplasm for the breeding of strawberry cultivars that help to counter the ongoing *D. suzukii* invasion.

## Materials and Methods

### *Fragaria* Accessions Analyzed

We analyzed 107 *Fragaria* accessions belonging to 12 species during an initial screen in 2015. The species dominating this study was *Fragaria vesca*, represented by 50 accessions, followed by 19 *Fragaria moschata*, 14 *Fragaria viridis*, nine *Fragaria chiloensis*, five *Fragaria nilgerrensis*, three *Fragaria* × *bifera*, and two *Fragaria orientalis* accessions. Five species—*Fragaria tibetica, Fragaria moupinensis, Fragaria virginiana, Fragaria cascadensis*, and *Fragaria nipponica*—were represented by a single accession.

**FIGURE 1:**
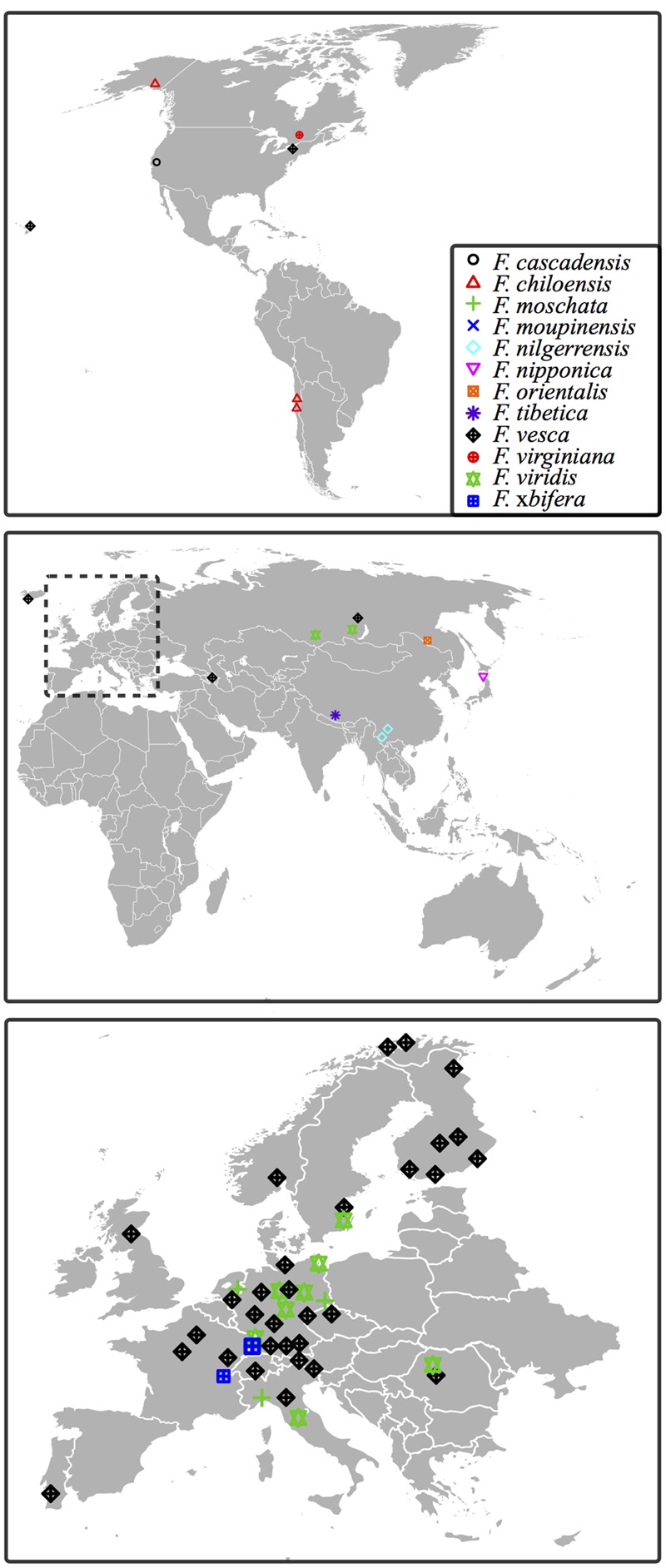
**Geographical origin of *Fragaria* accessions of the ‘Professor Staudt Collection’ tested in this study.** Geographical origins for the accessions are marked in each map: North and South America **(top)**; Africa, Asia and Australia **(middle)**; Europe **(bottom)**. Accessions with untraceable origins are not indicated. Accessions of one species collected from the same location are presented as a single label (see details in Supplementary Table S1).

Supplementary Table S1 lists information pertaining to each of the tested accession. These accessions comprise cultivars and worldwide collections, most of them from Europe (**Figure 1**). Among them, *F. vesca tetra* and *F. vesca poly 3* are artificial tetraploid descendants from diploid *F. vesca* ssp. *vesca* obtained *via* mutation breeding (Olbricht et al., 2014).

All *Fragaria* accessions analyzed in 2015 were maintained by Hansabred GmbH & Co. KG in Dresden, Germany. For each accession, at least three plants, which had been clonally propagated from stolons, were grown in a mixture of peat and sand in clay pots (20 cm in diameter) in a frost-free glasshouse in Dresden. The harvest and subsequent screening took place between late May and early July 2015. Ripe berries with the pedicel attached were carefully harvested between 06:00 and 08:00 h (UTC+2), packaged in soft tissue to prevent transportation damage and immediately sent to Munich, Germany, where the fruits were exposed to *D. suzukii* on the same day. All fruits that were bruised or damaged during transportation were excluded from further experiments.

Eighteen accessions were chosen to be analyzed again in 2016 (Supplementary Table S2). These accessions were maintained in the glasshouse (16 h:8 h, light:dark; 15–22°C; 60% humidity) at the Biocenter of the LMU Munich, Germany. Strawberry flowers were hand-pollinated and ripe strawberries were harvested between 14:00 and 15:00 h (UTC +2). Fruits were immediately used in the infestation assays.

The widest diameter of the fruit, as a representation of the fruit size, was measured using a size chart displaying circles with diameters ranging from 9 to 39 mm in 1 mm steps. The same fruits were then tested in the infestation assay (see below). For 9 out of 681 fruits in year 2015, the diameters were smaller than 9 mm. These outliers were not included in statistical analysis concerning fruit diameter. To accommodate all possible fruit diameters in 2016, circles with diameters ranging from 5 to 39 mm in 1 mm steps were used.

### Drosophila suzukii

The *D. suzukii* line used in this study was initially established by Nicolas Gompel from a single female collected in the French Alpes-Maritimes (43°52′45″N 7°26′34″E) in July 2011. The flies were maintained and expanded at 22°C on standard cornmeal medium in a climate chamber (50% humidity; 12 h:12 h, light:dark; light from 08:00 h). The infestation assays were carried out with 5- to 8-day-old fertilized *D. suzukii* females (Supplementary Method S1), which were isolated under brief anesthesia on a chill table (BioQuip Products Inc., Compton, CA, USA), and allowed to recover for 5 h at room temperature. An independent population of female *D. suzukii* was generated for each infestation assay.

### Infestation Assay

**FIGURE 2:**
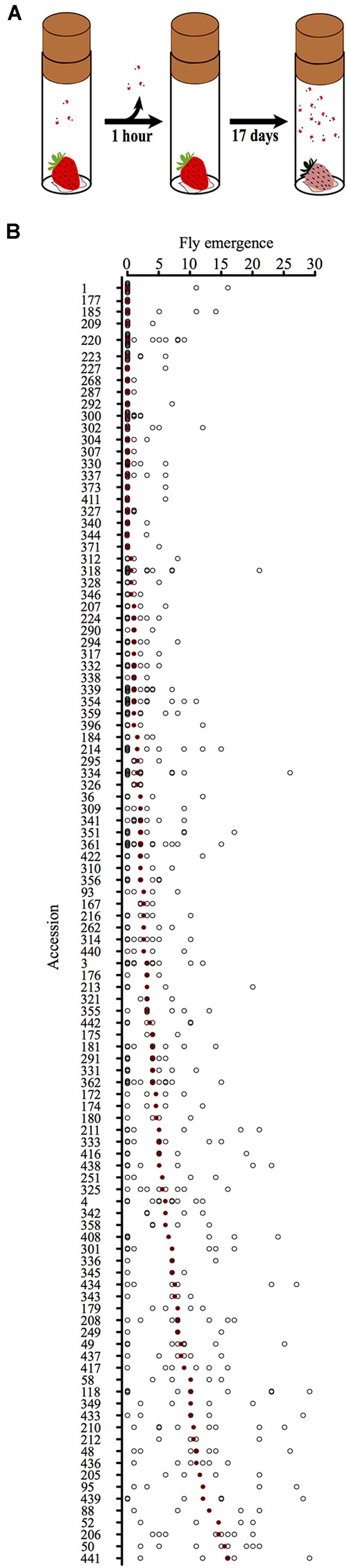
**(A)** Schematic drawing of the infestation assay. A single strawberry was placed on a wet tissue and enclosed with three female *D. suzukii* in a vial for defined incubation times. After the female flies were taken out of the vial, the strawberry was incubated for 17 days, during which time the number of emerging adult *D. suzukii* was determined (fly emergence). **(B)** Fly emergence of individual strawberries sorted by accessions. Black circles, number of flies emerging from single strawberries. Red dots, median values for the accessions.

Single strawberries were placed in separate polypropylene vials (28 mm × 85 mm; Semadeni, Ostermundigen, Germany) with a crumpled piece of tissue paper (125 mm × 125 mm) filling the bottom (**Figure 2A**). The tissue was soaked with 3 ml of Milli-Q filtered water, and vials were closed with Rayon foam stoppers (28 mm; K-TK, Retzstadt, Germany). Three *D. suzukii* females were transferred into each vial and incubated with the strawberry between 16:00 and 17:00 h in 2015, or 15:00 and 17:00 h (UTC +2) in 2016. The exposure time was therefore 1 and 2 h in 2015 and 2016, respectively. The time to adult fly emergence was measured in days post-exposure (DPE), with day 1 being the day of exposure itself. The vials were kept in the same climate chamber in which the flies had been maintained until 17 DPE. Emergence of adult flies was checked once per day. Fly emergence was defined as the total number of *D. suzukii* adults that emerged from an individual fruit until 17 DPE. Additionally, the number of eggs deposited into the individual fruits was determined at 2 DPE in 2016, except for the white fruits from accessions 214, 220 and 223, the color of which prevented accurate determination of egg numbers.

The infestation assays were conducted once per week for five successive weeks in 2015. At least four fruits were tested per accession for 102 of the 107 tested accessions. For the remaining five accessions, the limited fruit production only permitted analysis of three fruits (Supplementary Table S1). Four infestation assays were conducted in three successive weeks in 2016. In both years, each accession was tested in a variable number of independent infestation assays (Supplementary Tables S1 and S2).

## Results

### Fly Emergence Varies Strongly between Strawberry Accessions

Because soft fruits are the site of oviposition and larval development, we tested how well *D. suzukii* propagated in a total of 681 fruits representing 107 accessions of 12 *Fragaria* species. We exposed ripe fruits to a controlled number of fertilized *D. suzukii* female flies and counted the number of adult flies emerging from each berry (**Figure 2A**; see Materials and Methods).

The offspring typically needed 13–17 days to emerge. Rare cases of fly emergence beyond 17 DPE were observed (10/681 fruits), however, this late emergence phenomenon was not consistently observed in any particular accession. At 17 DPE, fungal growth was visible on all strawberries, yet with a variable extent of mycelium growth. No visually significant correlation between fungal growth and fly emergence was observed.

**Figure 2B** shows the fly emergence from individual strawberries sorted by accession. The median value of fly emergence for the accessions ranged from 0 to 16, indicating strong variation between the accessions tested.

To test whether the variation of fly emergence observed between berries correlated with their accession or their species, or both, we applied generalized linear mixed models (GLMMs; Supplementary Method S2; McCullagh and Nelder, 1989; Pinheiro and Bates, 2000; Bates et al., 2015; R Core Team, 2015; Supplementary Figures S2A,C). The accessions and species of the berry were both significant factors contributing to the emergence probability of a given accession (GLMM likelihood profile analysis, *P* < 0.01).

The data were collected from experiments carried out on five different days within a period of 5 weeks. We therefore tested whether the observed variation in emergence was due to uncontrolled variables that influenced the outcome at different days of exposure, such as variation in female fly oviposition behavior on different days. When we added the date of the individual experiment as a co-factor to the GLMM, it was not significant, but the species and the accession still were (Supplementary Method S2; *P* < 0.01), demonstrating that harvest times had no influence on the emergence probability.

Altogether, this dataset unveils a tractable range of variation in *D. suzukii* capability to emerge from the berries across the genus *Fragaria*.

### Variation in the Fly Emergence Correlates with Accessions and Fruit Diameter

Considering that major developmental steps of *D. suzukii* larvae take place inside the fruits, the developmental process might be influenced by the amount of available food (strawberry tissue) or physical conditions, or both, thus influencing the overall fly emergence from a berry. We therefore tested whether the fruit size, represented by the diameter of a fruit, was linked to fly emergence.

**FIGURE 3:**
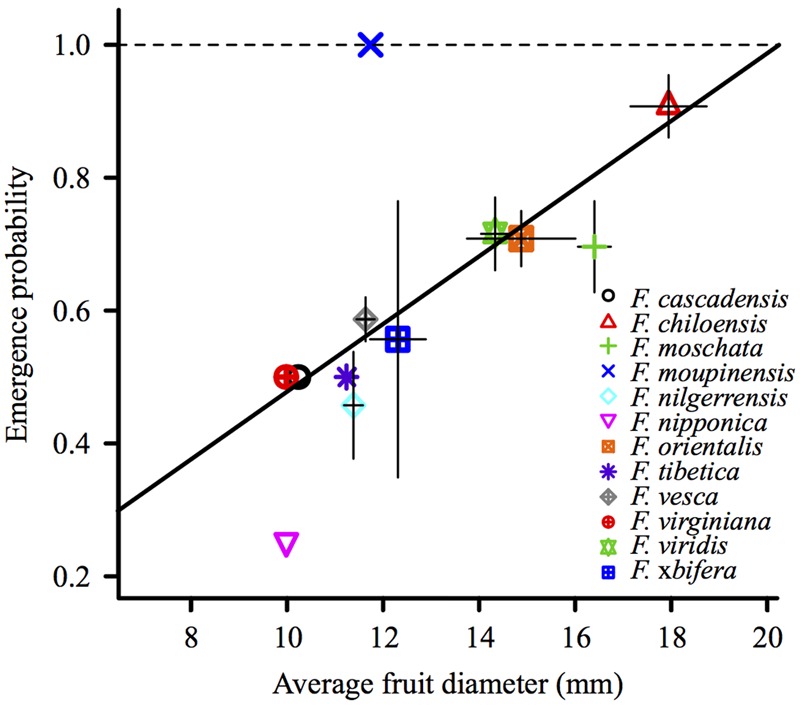
**Emergence probability correlates positively with the average fruit diameter of *Fragaria* species (*P* = 0.024).** Average fruit diameter of a species and average emergence probability were first averaged among the fruits of an accession, and then among the accessions of a species.

The diameter of individual berries ranged from 9 to 29 mm, and variations were observed among the individual fruits of each species (Supplementary Figure S1) and the average fruit diameter of accessions (Supplementary Table S1). We observed a significant positive correlation between the average emergence probability and average fruit diameter of *Fragaria* species (*P* = 0.024; **Figure 3**).

When accession, species and fruit diameter were analyzed in a single GLMM, the significance of the species vanished, whereas the accession number and fruit diameter remained significant factors (*P* < 0.001) for the emergence probability.

### Low Emergence Probability is Not Correlated with *D. suzukii*’s Proposed Center of Origin

The geographical origins of the tested accessions are scattered in several continents, including East Asia, from where *D. suzukii* originates (**Figure 1**). Due to the possible co-evolution between *Fragaria* species and *D. suzukii*, we initially suspected genotypes resulting in low fly emergence to also originate from East Asia. Whenever geographical information about the accessions (i.e., country of origin, longitude, latitude, or the distance to the equator) was added to the GLMM model (Supplementary Method S2), however, no significant effect was discovered.

### Identification of Candidate Accessions for the Breeding of Strawberry Cultivars That Limit the Proliferation of *D. suzukii*

We estimated the effect of accessions on emergence probability as well as fly emergence in separate GLMMs. In both cases we corrected for possible effects of fruit diameter. Ten accessions were predicted to have the strongest aversive effects in at least one model (Supplementary Figures S2B,C). These accessions were considered the candidate accessions with reduced fly emergence and were selected to be tested again in 2016, the year following the initial screen.

Five accessions that represented the other extreme end of the fly emergence and emergence probability, i.e., showing highest values of both traits, in the 2015 screen were included as controls (hereafter referred to as reference accessions). These reference accessions had similar fruit diameters as, and could be crossed with, the candidate accession belonging to the same species (Supplementary Table S2), thus representing potential crossing parents for positional cloning approaches to identify genes or QTLs determining fly emergence. We could not find suitable reference accession for accessions 1 and 118. Additionally, we re-tested three accessions, 327, 337 and 330, that had shown a low fly emergence, but were not among the top 10 candidates in the models (**Figure 2**; Supplementary Figures S2A,B; Supplementary Table S2). We excluded the accessions 1, 411 and 177 from the re-test analysis, due to limitations in fruit production.

In our experiments in 2016, we increased the number of fruits for each accession and counted the number of eggs deposited into individual fruits (other than those of accessions 214, 220 and 223). Fruits devoid of eggs (10.1%; 17/168) were excluded from further determination of fly emergence and emergence probability (Supplementary Figure S3; Supplementary Table S2). The re-test experiments of 18 accessions resulted in similar overall variation in fly emergence and emergence probability that had been observed in the initial 2015 screen (Supplementary Figure S4; Supplementary Table S2).

Of the five accessions that showed high fly emergence and emergence probability in 2015, only three, 206, 214 and 349, performed consistently in both years (Supplementary Figure S4; Supplementary Table S2), whereas 436 and 441 did not show similar fly emergence compared to 2015. Three candidate accessions, 223, 220 and 300, reproduced the low fly emergence phenotype, while other candidate accessions (185, 209, 118 and 340) failed to do so (Supplementary Figure S4). Similarly, only candidate accessions 300 and 340 showed relatively low emergence probability whereas other candidates did not (Supplementary Table S2).

Amongst the confirmed candidates, accession 300 was of particular interest. The number of eggs deposited into berries of accession 300 was not statistically different from that of the reference accession of *F. vesca*, 349 (Supplementary Figure S3; paired *t*-test, *P* > 0.05). However, only 30% of fruits of accession 300 supported fly emergence, a rate significantly lower than in accession 349 which had over 90% of its fruits supporting fly emergence (Supplementary Table S2; Fisher’s exact test, *P* < 0.005). In the few cases where *D. suzukii* did emerge from fruits of accession 300, a maximum of two flies emerged, also significantly fewer than from accession 349 (permutation test, *P* < 0.05), consistent with the phenotype of low fly emergence observed in 2015 (**Figure 2**; Supplementary Figure S4).

Overall, we found three accessions with reproducibly reduced fly emergence, of which accession 300 is particularly strong in countering fly emergence.

## Discussion

In response to the current outbreaks of *D. suzukii* worldwide, we initiated a search for lines that limit the fly propagation in their fruits, thus helping in reducing the spread of the pest. We tested 107 strawberry (genus *Fragaria*) accessions and discovered significant variations in the emergence of *D. suzukii* between these accessions. This variation in fly emergence correlated with the accession and fruit diameter. Fruit diameter was also correlated with species, demonstrating a major link between fruit size and the fly emergence from an accession. The fact that fly emergence was correlated with fruit size points to potential restrictions of space or resources imposed by the berry size. Although it is technically impossible to measure the sugar concentration and acidity value of individual fruits that at the same time are subject to the infestation assay, we have measured the sugar concentration (represented as brix value) and titratable acidity from accessions (Supplementary Method S3) and discovered no significant correlation to fly emergence, indicating that sugar or acidity value alone might not be the limiting factor for fly emergence.

Importantly, 10 accessions exhibited emergence probability, or fly emergence, or both, lower than that expected based on their fruit size. This important result suggests that the genetic make-up of these 10 accessions is responsible for the aversive effect on fly infestation. Re-testing of these 10 accessions has confirmed the low emergence probability and fly emergence phenotype of some but not all accessions (see Results). Accession 300 showed consistently low fly emergence and the majority of its fruits did not support the proliferation of *D. suzukii* in different years, strongly supporting that its fruits impose negative effects on *D. suzukii* development.

Our work explored the natural variation across the strawberry accessions in their ability to suppress *D. suzukii* emergence, providing a unique entry point into understanding the genetic basis of the fruit—*D. suzukii* interaction, which is currently poorly investigated. It is of our great interest to identify the responsible genes; and, the genotypes that confer reduced infestation rate are a valuable starting point for breeding strawberry cultivars that are less supportive of *D. suzukii* propagation, thus helping to reduce the damage caused by this pest.

## Author Contributions

KO, NG, and MP planned and designed the research. NB, KO and LB prepared experimental material. CP, LB, SZ, and XG performed experiments and collected the data. DM carried out the statistical analyses of the data. XG, MP and NG wrote the manuscript.

## Conflict of Interest Statement

The authors declare that the research was conducted in the absence of any commercial or financial relationships that could be construed as a potential conflict of interest.

The reviewer JL and handling Editor declared their shared affiliation, and the handling Editor states that the process nevertheless met the standards of a fair and objective review.

## References

[B1] Adrion J. R. Kousathanas A. Pascual M. Burrack H. J. Haddad N. M. Bergland A. O. (2014). *Drosophila suzukii*: the genetic footprint of a recent, worldwide invasion. *Mol. Biol. Evol.* 31 3148–3163. 10.1093/molbev/msu246 25158796 PMC4245814

[B2] Atallah J. Teixeira L. Salazar R. Zaragoza G. Kopp A. (2014). The making of a pest: the evolution of a fruit-penetrating ovipositor in *Drosophila suzukii* and related species. *Proc. R. Soc. B Biol. Sci.* 281:20132840 10.1098/rspb.2013.2840 PMC3953835 24573846

[B3] Bates D. Mächler M. B. Bolker B. Walker S. C. (2015). Fitting linear mixed-effects models using lme4. *J. Stat. Softw.* 67 1–48.

[B4] Berry J. A. (2012). *Pest Risk Assessment: Drosophila suzukii: Spotted wing Drosophila (Diptera: Drosophilidae) on Fresh Fruit From the USA*. Wellington: Ministry for Primary Industries.

[B5] Bolda M. P. Goodhue R. E. Zalom F. G. (2010). *Spotted wing Drosophila: Potential Economic Impact of a Newly Established Pest*. Berkeley CA: University of California 13 5–8.

[B6] Burrack H. J. Fernandez G. E. Spivey T. Kraus D. A. (2013). Variation in selection and utilization of host crops in the field and laboratory by *Drosophila suzukii* Matsumara (Diptera: Drosophilidae), an invasive frugivore. *Pest Manag. Sci.* 69 1173–1180. 10.1002/ps.3489 23494939

[B7] Calabria G. Máca J. Bächli G. Serra L. Pascual M. (2012). First records of the potential pest species *Drosophila suzukii* (Diptera: Drosophilidae) in Europe. *J. Appl. Entomol.* 136 139–147. 10.1111/j.1439-0418.2010.01583.x

[B8] Chabert S. Allemand R. Poyet M. Eslin P. Gibert P. (2012). Ability of European parasitoids (Hymenoptera) to control a new invasive Asiatic pest, *Drosophila suzukii*. *Biol. Control* 63 40–47. 10.1016/j.biocontrol.2012.05.005

[B9] Cini A. Anfora G. Escudero-Colomar L. A. Grassi A. Santosuosso U. Seljak G. (2014). Tracking the invasion of the alien fruit pest *Drosophila suzukii* in Europe. *J. Pest Sci.* 87 559–566. 10.1007/s10340-014-0617-z

[B10] Cini A. Ioriatti C. Anfora G. (2012). A review of the invasion of *Drosophila suzukii* in Europe and a draft research agenda for integrated pest management. *Bull. Insectol.* 65 149–160.

[B11] Cuthbertson A. G. S. Collins D. A. Blackburn L. F. Audsley N. Bell H. A. (2014). Preliminary screening of potential control products against *Drosophila suzukii*. *Insects* 5 488–498. 10.3390/insects5020488 26462696 PMC4592600

[B12] Deprá M. Poppe J. L. Schmitz H. J. De Toni D. C. Valente V. L. S. (2014). The first records of the invasive pest *Drosophila suzukii* in the South American continent. *J. Pest Sci.* 87 379–383. 10.1007/s10340-014-0591-5

[B13] Goodhue R. E. Bolda M. Farnsworth D. Williams J. C. Zalom F. G. (2011). Spotted wing drosophila infestation of California strawberries and raspberries: economic analysis of potential revenue losses and control costs. *Pest Manag. Sci.* 67 1396–1402. 10.1002/ps.2259 21815244

[B14] Hauser M. (2011). A historic account of the invasion of *Drosophila suzukii* (Matsumura) (Diptera: Drosophilidae) in the continental United States, with remarks on their identification. *Pest Manag. Sci.* 67 1352–1357. 10.1002/ps.2265 21898759

[B15] Haye T. Girod P. Cuthbertson A. G. S. Wang X. G. Daane K. M. Hoelmer K. A. (2016). Current SWD IPM tactics and their practical implementation in fruit crops across different regions around the world. *J. Pest Sci.* 89 643–651. 10.1007/s10340-016-0737-8

[B16] Hirakawa H. Shirasawa K. Kosugi S. Tashiro K. Nakayama S. Yamada M. (2013). Dissection of the octoploid strawberry genome by deep sequencing of the genomes of *Fragaria* species. *DNA Res.* 21 169–181. 10.1093/dnares/dst049 24282021 PMC3989489

[B17] Hummer K. E. Bassil N. Njuguna W. (2011). “*Fragaria*,” in *Wild Crop Relatives: Genomics and Breeding Resources* ed. Kole C. (Berlin: Springer-Verlag) 17–44.

[B18] Ioriatti C. Walton V. Dalton D. Anfora G. Grassi A. Maistri S. (2015). *Drosophila suzukii* (Diptera: Drosophilidae) and its potential impact to wine grapes during harvest in two cool climate wine grape production regions. *J. Econ. Entomol.* 108 1148–1155. 10.1093/jee/tov042 26470240

[B19] Kaneshiro K. Y. (1983). *Drosophila* (Sophophora) suzukii (Matsumura). *Proc. Hawaiian Entomol. Soc.* 24 179.

[B20] Kanzawa T. (1936). Studies on *Drosophila suzukii* Mats. *J. Plant Prot.* 23 66–70.

[B21] Kinjo H. Kunimi Y. Ban T. Nakai M. (2013). Oviposition efficacy of *Drosophila suzukii* (Diptera: Drosophilidae) on different cultivars of blueberry. *J. Econ. Entomol.* 106 1767–1771. 10.1603/EC12505 24020291

[B22] Lee J. C. Bruck D. J. Curry H. Edwards D. Haviland D. R. Van Steenwyk R. A. (2011). The susceptibility of small fruits and cherries to the spotted-wing drosophila, *Drosophila suzukii*. *Pest Manag. Sci.* 67 1358–1367. 10.1002/ps.2225 21710685

[B23] Lee J. C. Dalton D. T. Swoboda-Bhattarai K. A. Bruck D. J. Burrack H. J. Strik B. C. (2015). Characterization and manipulation of fruit susceptibility to *Drosophila suzukii*. *J. Pest Sci.* 89 771–780. 10.1007/s10340-015-0692-9

[B24] Lin Q. Zhai Y. Zhang A. Men X. Zhang X. Zalom F. G. (2014). Comparative developmental times and laboratory life tables for *Drosophila suzukii* and *Drosophila* melanogaster (Diptera: Drosophilidae). *Fla. Entomol.* 97 1434–1442. 10.1653/024.097.0418

[B25] McCullagh P. Nelder J. A. (1989). *Generalized Linear Models.* Boca Raton, FL: CRC Press.

[B26] Olbricht K. Kallweit L. Mannicke F. Drewes-Alvarez R. Vogt R. (2014). The *Fragaria* Herbarium of Professor Günter Staudt. *Acta Hortic.* 1049 305–308. 10.17660/ActaHortic.2014.1049.40

[B27] Pinheiro J. C. Bates D. M. (2000). *Mixed-effects models in S and S-PLUS*. New York, NY: Springer.

[B28] R Core Team (2015). *R: A Language Environment for Statistical Computing*. Vienna: R Foundation for Statistical Computing.

[B29] Shulaev V. Sargent D. J. Crowhurst R. N. Mockler T. C. Folkerts O. Delcher A. L. (2011). The genome of woodland strawberry (*Fragaria* vesca). *Nat. Genet.* 43 109–116. 10.1038/ng.740 21186353 PMC3326587

[B30] Walsh D. B. Bolda M. P. Goodhue R. E. Dreves A. J. Lee J. Bruck D. J. (2011). *Drosophila suzukii* (Diptera: Drosophilidae): invasive pest of ripening soft fruit expanding its geographic range and damage potential. *J. Integr. Pest Manag.* 2 1–7. 10.1603/IPM10010

