# Supplementary material for: Strawberry Accessions with Reduced Drosophila suzukii Emergence From Fruits
Source: Front Plant Sci. 2016 Dec 21;7:1880. doi: 10.3389/fpls.2016.01880 (PMC5174125; doi:10.3389/fpls.2016.01880)
Supplement: Supplementary file 1 [file Table_1.PDF]

Table S1. Detailed information of the 107 *Fragaria* accessions tested in 2015.

Acce -ssion	Species	Ploidy	Repeti -tion(s)	N	Fruit diameter (mm)	Fly emergence (median)	Origin (country)	Geographical coordinates	Subspecies/ Cultivar
**1**	*F.×bifera*	2x	2	14	13 ± 1	0.0	Germany	47°49'18.9"N 7°39'04.3"E	
**3**	*F.×bifera*	2x	2	11	11 ± 1	3.0	France	47°17'06.1"N 6°02'34.8"E	
**4**	*F.×bifera*	2x	2	10	13 ± 1	6.0	Germany	49°54'52.7"N 11°26'30.7"E	
**36**	*F. cascadensis*	10x	1	4	10 ± 1	2.0	USA	44°22'14.3"N 121°51'56.2"W	
**48**	*F. chiloensis*	8x	2	7	17 ± 1	11.0	USA	60°32'41.0"N 145°45'44.0"W	ssp. *pacifica*
**49**	*F. chiloensis*	8x	2	8	19 ± 2	8.5	USA	60°32'41.0"N 145°45'44.0"W	ssp. *pacifica*
**50**	*F. chiloensis*	8x	2	8	20 ± 1	15.5	USA	60°32'41.0"N 145°45'44.0"W	ssp. *pacifica*
**52**	*F. chiloensis*	8x	1	4	19 ± 2	14.5	USA	60°32'41.0"N 145°45'44.0"W	ssp. *pacifica*
**58**	*F. chiloensis*	8x	1	4	14 ± 1	10.0	Chile	39°38'12.9"S 71°30'11.4"W	ssp. *chiloensis* f. *patagonica*
**88**	*F. chiloensis*	8x	1	4	17 ± 2	13.0	Chile	38°22'52.0"S 71°35'13.5"W	ssp. *chiloensis* f. *patagonica*
**93**	*F. chiloensis*	8x	1	4	17 ± 1	2.5	Chile	38°22'52.0"S 71°35'13.5"W	ssp. *chiloensis* f. *patagonica*
**95**	*F. chiloensis*	8x	1	4	16 ± 1	12.0	Chile	38°22'52.0"S 71°35'13.5"W	ssp. *chiloensis* f. *patagonica*
**118**	*F. chiloensis*	8x	2	9	22 ± 4	10.0	N/A	N/A	Lucida Perfecta
**167**	*F. moupinensis*	4x	1	4	12 ± 2	2.5	Germany	51°49'39.8"N 11°18'19.2"E	
**172**	*F. moschata*	6x	1	4	18 ± 1	4.5	Germany	N/A	Schöne Wienerin
**174**	*F. moschata*	6x	1	4	15 ± 1	4.5	Germany	51°07'46.3"N 14°55'54.9"E	
**175**	*F. moschata*	6x	1	3	18 ± 1	4.0	France	N/A	Versaillaise
**176**	*F. moschata*	6x	1	3	18 ± 1	3.0	France	N/A	Capron Royal
**177**	*F. moschata*	6x	1	4	15 ± 3	0.0	N/A	N/A	Hautboy
**179**	*F. moschata*	6x	1	4	19 ± 2	8.0	N/A	N/A	Bauwens
**180**	*F. moschata*	6x	1	4	19 ± 1	4.5	N/A	N/A	Pekun
**181**	*F. moschata*	6x	2	8	15 ± 3	4.0	The Netherlands	50°49'16.2"N 5°41'08.9"E	
**184**	*F. moschata*	6x	1	4	16 ± 2	1.5	Italy	44°51'50.2"N 8°53'43.0"E	Profumata di Tortona
**185**	*F. moschata*	6x	1	8	15 ± 2	0.0	Italy	44°51'50.2"N 8°53'43.0"E	Profumata di Tortona, weibl. Casalini
**205**	*F. moschata*	6x	1	4	18 ± 3	11.5	Germany	49°77'69.82"N 9°96'55.49"E	
**206**	*F. moschata*	6x	2	8	16 ± 1	14.5	Germany	49°77'69.82"N 9°96'55.49"E	
**207**	*F. moschata*	6x	1	4	16 ± 2	1.0	Germany	49°77'69.82"N 9°96'55.49"E	
**208**	*F. moschata*	6x	2	8	16 ± 3	8.0	Germany	49°77'69.82"N 9°96'55.49"E	
**209**	*F. moschata*	6x	2	7	16 ± 3	0.0	Germany	49°77'69.82"N 9°96'55.49"E	
**210**	*F. moschata*	6x	2	8	17 ± 1	10.5	Germany	49°77'69.82"N 9°96'55.49"E	
**211**	*F. moschata*	6x	1	6	16 ± 0	5.0	Germany	49°77'69.82"N 9°96'55.49"E	
**212**	*F. moschata*	6x	1	4	15 ± 1	10.5	Germany	49°77'69.82"N 9°96'55.49"E	
**213**	*F. moschata*	6x	1	4	15 ± 2	3.0	Germany	49°77'69.82"N 9°96'55.49"E	
**214**	*F. nilgerrensis*	2x	2	10	11 ± 1	1.5	China	N/A	
**216**	*F. nilgerrensis*	2x	1	6	11 ± 1	2.5	N/A	N/A	Apricot Chinoise
**220**	*F. nilgerrensis*	2x	3	22	11 ± 1	0.0	China	N/A	
**223**	*F. nilgerrensis*	2x	3	13	12 ±1	0.0	China	26°23'01.8"N 108°12'26.3"E	
**224**	*F. nilgerrensis*	2x	2	7	11 ± 1	1.0	China	26°23'01.8"N 108°12'26.3"E	
**227**	*F. nipponica*	2x	1	4	10 ± 1	0.0	Japan	N/A	ssp. *nipponica*
**249**	*F. orientalis*	4x	1	3	16 ± 1	8.0	Russia	N/A	
**251**	*F. orientalis*	4x	1	4	14 ± 1	5.5	Russia	N/A	
**262**	*F. tibetica*	4x	1	4	11 ± 1	2.5	Tibet (China)	N/A	
**268**	*F. vesca*	2x	1	4	11 ± 2	0.0	USA	44°27'05.4"N 71°34'42.2"W	ssp. *americana*
**287**	*F. vesca*	2x	1	4	10 ± 0	0.0	Germany	47°34'42.8"N 10°03'15.7"E	ssp. *vesca*
**290**	*F. vesca*	2x	1	3	10 ± 1	1.0	Russia	N/A	ssp. *vesca*
**291**	*F. vesca*	2x	2	7	11 ± 1	4.0	Germany	47°48'11.9"N 8°02'12.8"E	ssp. *vesca*
**292**	*F. vesca*	2x	1	4	12 ±1	0.0	Cech Republic	50°53'00.3"N 14°16'53.9"E	ssp. *vesca*
**294**	*F. vesca*	2x	2	7	12 ± 2	1.0	France	48°25'16.5"N 7°41'00.3"E	ssp. *vesca*
**295**	*F. vesca*	2x	1	4	10 ± 1	1.5	Finland	61°13'28.6"N 28°06'21.3"E	ssp. *vesca*
**300**	*F. vesca*	2x	3	15	10 ± 1	0.0	Germany	50°41'49.3"N 13°07'08.5"E	ssp. *vesca*
**301**	*F. vesca*	2x	2	6	13 ± 1	7.5	Norway	59°54'56.5"N 10°45'05.8"E	ssp. *vesca*
**302**	*F. vesca*	2x	2	8	13 ± 1	0.0	USA	N/A	f. *semperflorens*
**304**	*F. vesca*	2x	2	5	10 ± 1	0.0	Iceland	64°08'23.0"N 21°52'09.3"W	ssp. *vesca*
**307**	*F. vesca*	2x	1	4	10 ± 1	0.0	Austria	47°13'40.4"N 13°09'16.2"E	ssp. *vesca*

Repetitions, number of independent experiments; N, number of fruits tested; Fruit diameter, average ± SD; N/A, not available

Table S1 Continued.

Acce-ssion	Species	Ploidy	Repeti-tion(s)	N	Fruit diameter (mm)	Fly emergence (median)	Origin (country)	Geographical coordinates	Subspecies/ Cultivar
**309**	*F. vesca*	2x	1	4	11 ± 1	2.0	Austria	47°13'40.4"N 13°09'16.2"E	ssp. *vesca*
**310**	*F. vesca*	2x	1	4	12 ± 1	2.0	Austria	47°13'40.4"N 13°09'16.2"E	ssp. *vesca*
**312**	*F. vesca*	2x	1	4	10 ± 1	0.5	Azerbaijan	N/A	ssp. *vesca*
**314**	*F. vesca*	2x	2	8	13 ± 2	2.5	The Netherlands	50°52'18.8"N 5°39'48.8"E	ssp. *vesca*
**317**	*F. vesca*	2x	1	4	14 ± 1	1.0	Germany	51°10'21.5"N 13°39'26.5"E	f. *alba*
**318**	*F. vesca*	2x	3	14	11 ± 1	0.5	Sweden	62°34'29.2"N 12°17'23.3"E	ssp. *vesca*
**321**	*F. vesca*	2x	1	3	11 ± 1	3.0	Portugal	38°47'41.1"N 9°23'25.0"W	ssp. *vesca*
**325**	*F. vesca*	2x	2	8	14 ± 1	5.5	N/A	N/A	f. *semperflorens*; Reine des Vallées
**326**	*F. vesca*	2x	1	4	10 ± 1	1.5	Germany	51°45'21.6"N 10°50'58.7"E	ssp. *vesca*
**327**	*F. vesca*	2x	2	7	11 ± 2	0.0	Romania	46°46'08.2"N 23°35'12.9"E	ssp. *vesca*
**328**	*F. vesca*	2x	1	4	12 ± 1	0.5	Germany	47°40'35.6"N 7°52'26.7"E	ssp. *vesca*
**330**	*F. vesca*	2x	2	8	12 ± 2	0.0	UK	55°50'58.1"N 3°19'42.4"W	ssp. *vesca*
**331**	*F. vesca*	2x	2	7	12 ± 1	4.0	France	50°00'55.5"N 2°41'50.4"E	ssp. *vesca*
**332**	*F. vesca*	2x	2	8	14 ± 2	1.0	UK	55°59'27.3"N 3°24'07.2"W	f. *alba*
**333**	*F. vesca*	2x	2	8	11 ± 1	5.0	Sweden	56°37'50.4"N 16°27'21.3"E	ssp. *vesca*
**334**	*F. vesca*	2x	3	14	12 ± 1	1.5	Germany	N/A	f. *alba*
**336**	*F. vesca*	2x	1	3	13 ± 1	7.0	Italy	45°56'33.3"N 10°48'48.0"E	ssp. *vesca*
**337**	*F. vesca*	2x	2	8	11 ± 1	0.0	Italy	45°56'33.3"N 10°48'48.0"E	ssp. *vesca*
**338**	*F. vesca*	2x	1	4	11 ± 1	1.0	Germany	51°25'02.7"N 11°08'12.8"E	ssp. *vesca*
**339**	*F. vesca*	2x	2	14	12 ± 2	1.0	Germany	49°47'01.6"N 11°21'46.9"E	ssp. *vesca*
**340**	*F. vesca*	2x	1	4	14 ± 2	0.0	France	48°48'36.4"N 2°06'39.2"E	ssp. *vesca*
**341**	*F. vesca*	2x	3	9	12 ± 1	2.0	Germany	50°35'22.8"N 9°13'21.3"E	ssp. *vesca*
**342**	*F. vesca*	2x	1	4	14 ± 0	6.0	Germany	47°44'22.6"N 9°43'11.4"E	ssp. *vesca*
**343**	*F. vesca*	2x	1	4	14 ± 1	7.5	Switzerland	46°13'20.6"N 7°31'22.4"E	ssp. *vesca*
**344**	*F. vesca*	2x	1	4	11 ± 1	0.0	Germany	51°01'10.8"N 11°19'06.4"E	ssp. *vesca*
**345**	*F. vesca*	2x	1	3	11 ± 1	7.0	Austria	46°36'21.0"N 14°02'41.7"E	spp. *vesca*
**346**	*F. vesca*	2x	1	4	14 ± 1	7.0	Germany	49°46'12.87"N 9°93'09.24"E	ssp. *vesca*
**349**	*F. vesca*	2x	1	4	13 ± 2	10.0	N/A	N/A	f. *semperflorens*; Quarantaine de Prin
**351**	*F. vesca*	2x	1	9	14 ± 1	2.0	N/A	N/A	f. *semperflorens*; Yellow Wonder
**354**	*F. vesca*	2x	3	15	11 ± 1	1.0	Finland	60°29'12.2"N 22°09'12.2"E	spp. *vesca*
**355**	*F. vesca*	2x	2	8	12 ± 1	3.0	Finland	60°13'45.2"N 25°01'23.8"E	spp. *vesca*
**356**	*F. vesca*	2x	2	7	11 ± 2	2.0	Finland	61°34'56.8"N 26°01'07.2"E	spp. *vesca*
**358**	*F. vesca*	2x	1	4	13 ± 3	6.0	Finland	63°03'00.0"N 25°49'12.0"E	spp. *vesca*
**359**	*F. vesca*	2x	2	8	11 ± 1	1.0	Finland	67°47'31.2"N 29°26'49.6"E	spp. *vesca*
**361**	*F. vesca*	2x	3	15	11 ± 1	2.0	Norway	69°32'09.1"N 20°22'49.9"E	spp. *vesca*
**362**	*F. vesca*	2x	2	13	10 ± 1	4.0	Norway	70°10'01.5"N 24°45'22.0"E	spp. *vesca*
**371**	*F. vesca*	4x	1	4	12 ± 2	0.0	Germany	N/A	breeding
**373**	*F. vesca*	4x	1	4	11 ± 0	0.0	Germany	N/A	breeding
**396**	*F. virginiana*	8x	1	4	9 ± 2	1.0	Canada	45°55'38.2"N 74°07'31.1"W	
**408**	*F. viridis*	2x	1	6	17 ± 2	6.5	Germany	54°35'26.3"N 13°07'42.0"E	
**411**	*F. viridis*	2x	1	5	16 ± 1	0.0	Germany	51°01'27.8"N 13°42'10.1"E	
**416**	*F. viridis*	2x	1	6	14 ± 1	5.0	Russia	52°18'48.2"N 104°22'18.7"E	
**417**	*F. viridis*	2x	1	6	15 ± 1	9.0	Russia	52°18'48.2"N 104°22'18.7"E	
**422**	*F. viridis*	2x	1	5	13 ± 3	2.0	Germany	51°11'00.1"N 13°33'54.9"E	
**433**	*F. viridis*	2x	1	6	14 ± 2	10.0	Sweden	56°31'25.6"N 16°26'04.5"E	
**434**	*F. viridis*	2x	1	6	15 ± 2	7.5	Romania	46°40'48.0"N 22°39'05.0"E	
**436**	*F. viridis*	2x	1	6	13 ± 2	11.0	Germany	51°29'44.2"N 9°03'27.3"E	
**437**	*F. viridis*	2x	1	6	13 ± 1	8.5	Germany	49°46'37.1"N 9°57'56"E	
**438**	*F. viridis*	2x	1	6	14 ± 1	5.0	Germany	49°46'37.1"N 9°57'56"E	
**439**	*F. viridis*	2x	1	6	14 ± 1	12.0	Germany	49°46'37.1"N 9°57'56"E	
**440**	*F. viridis*	2x	1	4	15 ± 3	2.5	Germany	49°46'37.1"N 9°57'56"E	
**441**	*F. viridis*	2x	1	5	14 ± 1	16.0	Germany	49°46'37.1"N 9°57'56"E	
**442**	*F. viridis*	2x	1	6	13 ± 2	3.5	Germany	49°46'37.1"N 9°57'56"E	

Repetitions, number of independent experiments; N, number of fruits tested; Fruit diameter, average ± SD; N/A, not available
