# Supplementary material for: Strawberry Accessions with Reduced Drosophila suzukii Emergence From Fruits
Source: Front Plant Sci. 2016 Dec 21;7:1880. doi: 10.3389/fpls.2016.01880 (PMC5174125; doi:10.3389/fpls.2016.01880)
Supplement: Supplementary file 2 [file Table_2.pdf]

Table S2. Results obtained on the 15 *Fragaria* accessions re-tested in 2016. Open triangles, reference accessions that showed high fly emergence and emergence probability in 2015 and can be crossed with the candidate accessions of the same species. Closed triangles, candidate accessions identified from 2015.

Species	Acc.	N	Total # of fruits	# of fruits (# of eggs ≥ 1)	# of fruits (0 fly emergence in fruits with eggs)	Emergence probability
F. moschata	▷ 206	4	24	23	2	91.3%
	▶ 185	4	22	20	5	75.0%
	▶ 209	2	9	9	0	100.0%
F. vesca	▷ 349	4	17	15	1	93.3%
	▶ 300	4	23	20	14	30.0%
	▶ 340	2	9	9	3	66.7%
	327	4	19	18	5	72.2%
	330	4	7	7	3	57.1%
	337	4	23	20	7	65.0%
F. nilgerrensis	▷ 214	2	5	N/A	N/A	N/A
	▶ 220	2	5	N/A	N/A	N/A
	▶ 223	2	6	N/A	N/A	N/A
F. viridis	▷ 436	1	5	3	2	33.3%
	▷ 441	1	3	2	1	50.0%
F. chiloensis	▶ 118	3	7	5	0	100.0%

Acc., accession; #, number; N/A, not available; N, number of independent infestation assays
