# Supplementary material for: Strawberry Accessions with Reduced Drosophila suzukii Emergence From Fruits
Source: Front Plant Sci. 2016 Dec 21;7:1880. doi: 10.3389/fpls.2016.01880 (PMC5174125; doi:10.3389/fpls.2016.01880)
Supplement: Supplementary file 3 [file Presentation_1.PDF]

Method S1. Fertility test of female *Drosophila suzukii.* The fertility of females used in the infestation assay was ensured as follows: individual five to six-days old female *D. suzukii* that had been kept in a population with males until experimentation and were isolated into separate tubes containing standard cornmeal medium. The number of tube yielding progeny was scored a week later. Twenty-four out of 25 females produced progeny, indicating a fertilization rate ≥ 96% within the female population used for the infestation assays.

Method S2. Statistical analysis with generalized liner mixed models (GLMMs). Logistic regression with mixed effects, i.e., generalized linear mixed models (GLMMs) of the binomial type with logit-link (McCullagh & Nelder, 1989; Pinheiro & Bates, 2000), was applied to data obtained from year 2015 using the software package lme4 (version 1.1) for the R statistics software, version 3.1 (Bates *et al.*, 2015; R Core Team, 2015). Emergence probability was defined as the number of fruits showing emergence of at least one fly divided by the total number of fruits tested for this accession, and calculated for each accession. Emergence probability was analyzed in the GLMMs to determine whether it depends on the accession, species, the fruit diameter, or the geographical origin of the accession. Factors modeled as random effects were accession, species and country of origin, while the remaining factors were modeled as fixed effects. Both fly emergence and emergence probability were used in GLMMs to identify accessions that display low fly emergence and emergence probability, respectively.

Method S3. Measurement of soluble solids content (SSC in °Brix) and titratable acidity (TA in % of citric acid equivalent) in strawberries. All, but only fully ripe and typical fruits from one season of at least five plants per accession were harvested and stored at -20°C until fruit preparation. A representative batch of at least 50 fruits per accession were used in the measurement. The brix value of the fruit sap from thawed fruit was measured using a digital refractometer (Quick-Brix®, Mettler Toledo, Schwerzenbach, Germany). A homogenate of the batch produced with a household mixer (Bosch professional MSM 71.) for 2 min was used for the determination of TA using a titrator (716DMS-Titrino-Serie-06® (Metrohm, Filderstadt, Germany).
