# Supplementary material for: Strawberry Accessions with Reduced Drosophila suzukii Emergence From Fruits
Source: Front Plant Sci. 2016 Dec 21;7:1880. doi: 10.3389/fpls.2016.01880 (PMC5174125; doi:10.3389/fpls.2016.01880)
Supplement: Supplementary file 4 [file Image_1.pdf]

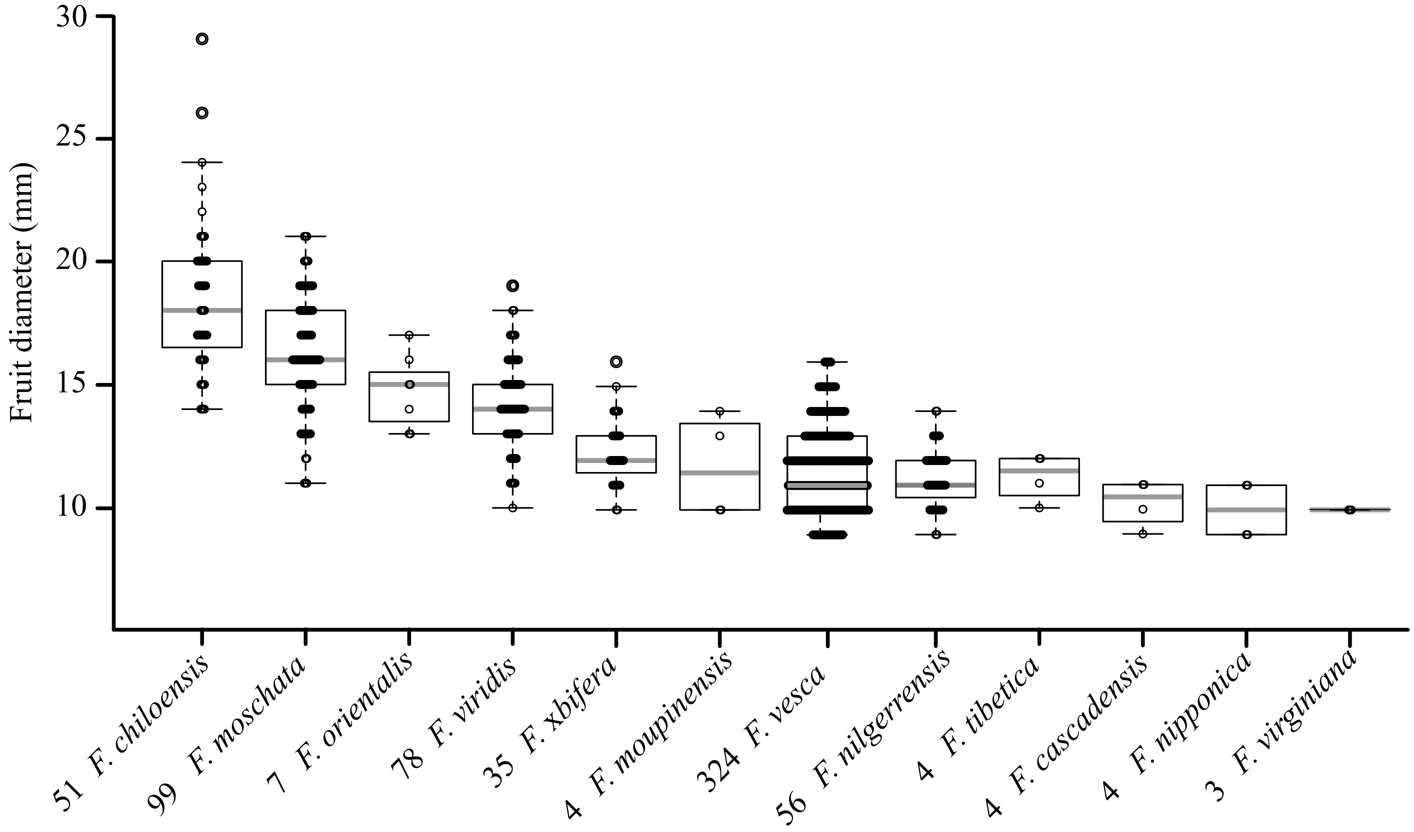

Figure S1. Fruit diameter of 12 *Fragaria* species. Numbers in front of the species names indicate the number of fruits analyzed. Black circles, the fruit diameters of individual strawberries. Grey lines, median values of fruit diameter of the species. The box plot follows standard patterns: the box presents 50% of all values; upper and lower whisker each presents 25% of all values.
