# Supplementary material for: Strawberry Accessions with Reduced Drosophila suzukii Emergence From Fruits
Source: Front Plant Sci. 2016 Dec 21;7:1880. doi: 10.3389/fpls.2016.01880 (PMC5174125; doi:10.3389/fpls.2016.01880)
Supplement: Supplementary file 5 [file Image_2.pdf]

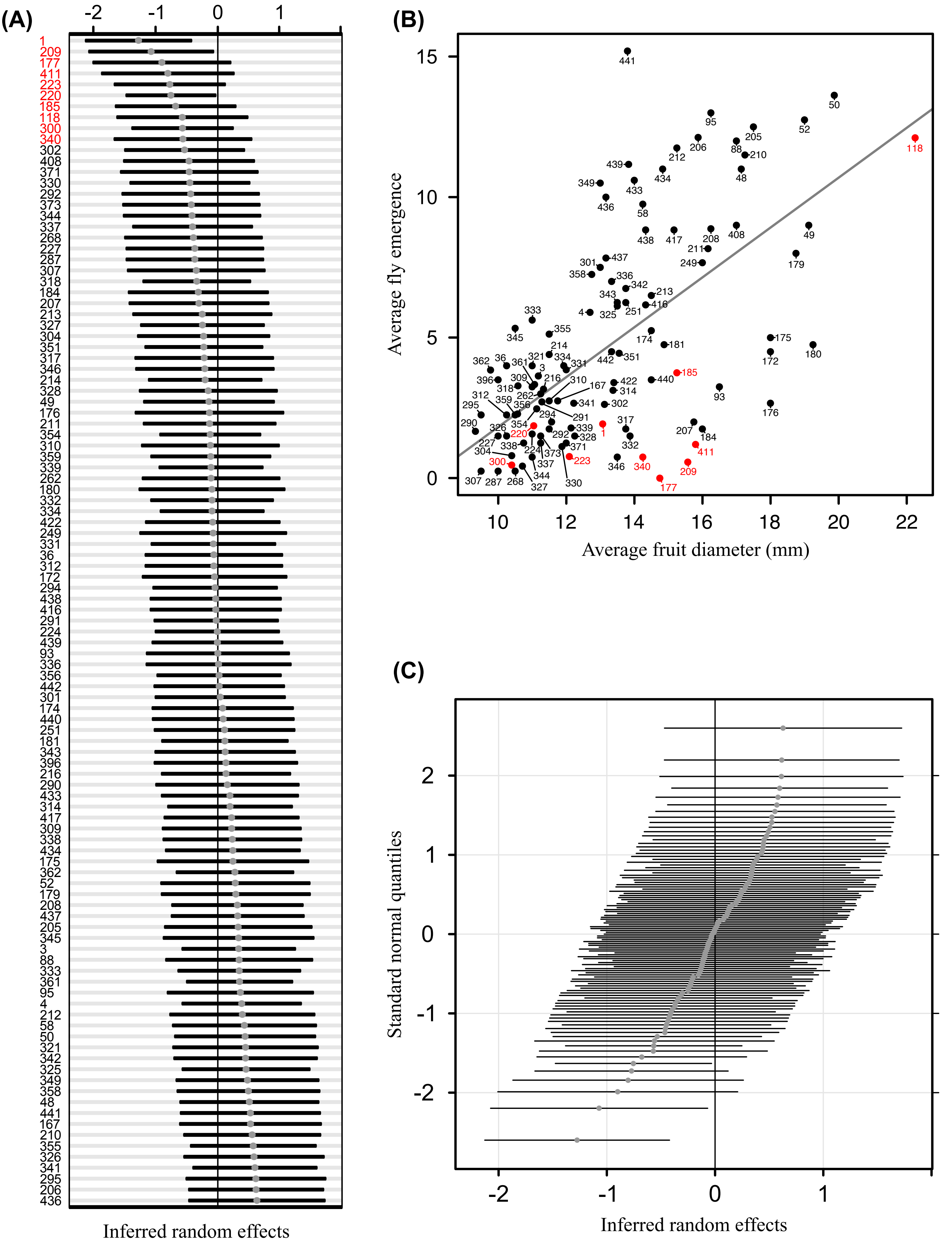
(A)
Inferred random effects
(B)
Average fly emergence
Average fruit diameter (mm)
(C)
Standard normal quantiles
Inferred random effects

Figure S2

Figure S2. Statistical analysis with GLMM to identify candidate accessions. (**A**) Inferred random effects of accessions on emergence probability are estimated in a logistic regression model (GLMM). The effect of fruit diameter is measured as a separate coefficient, thus the inferred effects shown here are given by the characters of the accession except its fruit diameter. Ten accessions showing strongest negative effects on emergence probability were tested again in 2016 and are denoted by red color. Grey dots, random effects for the accessions. The bars present 95% confidence interval (without multiple-testing correction). (**B**) The average fruit diameter was plotted against the average fly emergence of all 107 accessions, as an alternative graphical representation to the GLMM model that were fitted with average fruit diameter and fly emergence. Accessions marked with red are the same candidate accessions in (**A**). (**C**) Quantiles of the inferred random effects with standard error bars plotted against the normal distribution quantiles, indicating that the distribution of the random effects is sufficiently close to normality.
