# Supplementary material for: Strawberry Accessions with Reduced Drosophila suzukii Emergence From Fruits
Source: Front Plant Sci. 2016 Dec 21;7:1880. doi: 10.3389/fpls.2016.01880 (PMC5174125; doi:10.3389/fpls.2016.01880)
Supplement: Supplementary file 7 [file Image_4.pdf]

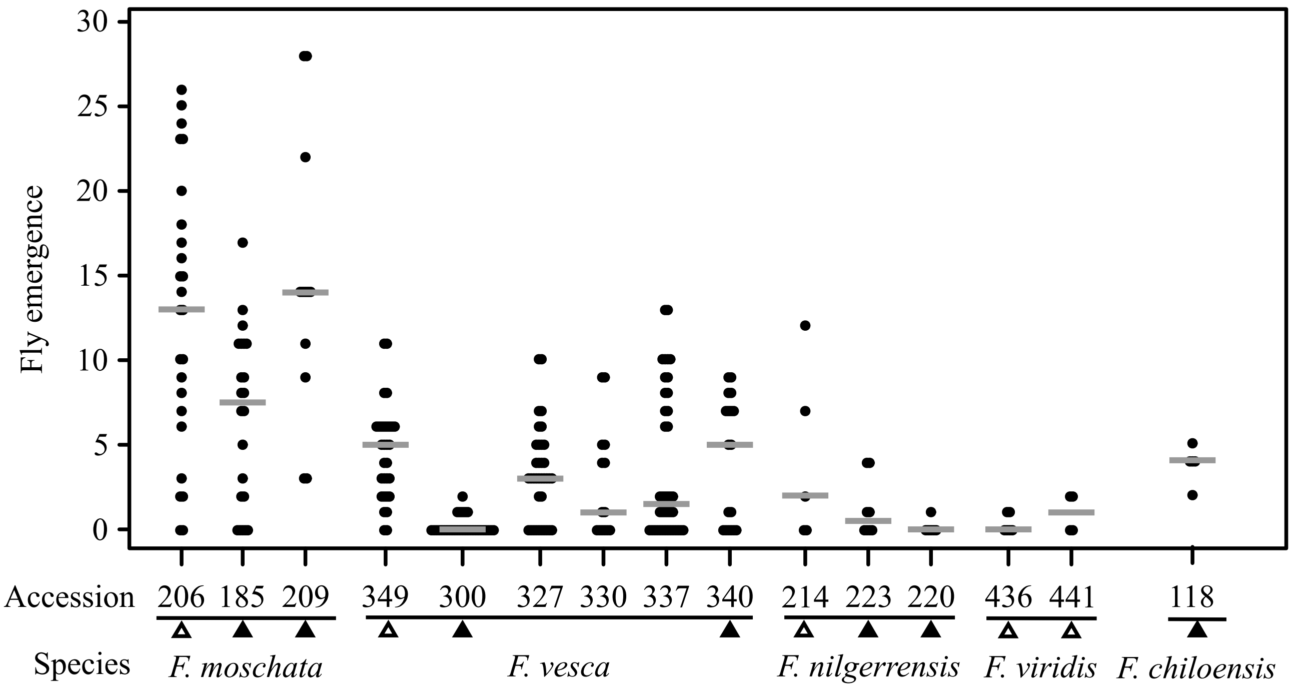

Figure S4. Fly emergence from individual fruits of 15 *Fragaria* accessions tested in 2016. Open triangles, reference accessions that showed high fly emergence and emergence probability in 2015 and can be crossed with the candidate accessions of the same species. Closed triangles, candidate accessions identified from 2015. Black dots, fly emergence from individual fruits of the accessions. Grey lines, median.
